# Characterisation of bioenergetic pathways and related regulators by multiple assays in human tumour cells

**DOI:** 10.1186/s12935-016-0281-x

**Published:** 2016-02-11

**Authors:** A. Jeney, Z. Hujber, N. Szoboszlai, A. Fullár, J. Oláh, É. Pap, Á. Márk, Cs. Kriston, J. Kralovánszky, I. Kovalszky, K. Vékey, A. Sebestyén

**Affiliations:** 1st Department of Pathology and Experimental Cancer Research, Semmelweis University, Üllői út 26, Budapest, 1085 Hungary; Laboratory of Environmental Chemistry and Bioanalytics, Department of Analytical Chemistry, Institute of Chemistry, Eötvös Loránd University, P.O. Box 32, Budapest, 1518 Hungary; Department of Clinical Research, National Institute of Oncology, P.O. Box 21, Budapest, 1525 Hungary; Research Centre for Natural Sciences of the Hungarian Academy of Sciences, Pusztaszeri u. 59-67, Budapest, 1025 Hungary; Tumour progression Research Group of Joint Research Organization of Hungarian Academy of Sciences, Semmelweis University, Budapest, Hungary

**Keywords:** Bioenergetic signature, Metabolic characterisation, Energy metabolism, Glucose/acetate utilization, GAPDH/β-F1-ATPase expression, TCA impairment, mTOR

## Abstract

**Background:**

Alterations in cellular metabolism are considered as hallmarks of cancers, however, to recognize these alterations and understand their mechanisms appropriate techniques are required. Our hypothesis was to determine whether dominant bioenergetic mechanism may be estimated by comparing the substrate utilisation with different methods to detect the labelled carbon incorporation and their application in tumour cells.

**Methods:**

To define the bioenergetic pathways different metabolic tests were applied: (a) measuring CO_2_ production from [1-^14^C]-glucose and [1-^14^C]-acetate; (b) studying the effect of glucose and acetate on adenylate energy charge; (c) analysing glycolytic and TCA cycle metabolites and the number of incorporated ^13^C atoms after [U-^13^C]-glucose/[2-^13^C]-acetate labelling. Based on [1-^14^C]-substrate oxidation two selected cell lines out of seven were analysed in details, in which the highest difference was detected at their substrate utilization. To elucidate the relevance of metabolic characterisation the expression of certain regulatory factors, bioenergetic enzymes, mammalian target of rapamycin (mTOR) complexes (C1/C2) and related targets as important elements at the crossroad of cellular signalling network were also investigated.

**Results:**

Both [U-^13^C]-glucose and [1-^14^C]-substrate labelling indicated high glycolytic capacity of tumour cells. However, the ratio of certain ^13^C-labelled metabolites showed detailed metabolic differences in the two selected cell lines in further characterisation. The detected differences of GAPDH, β-F1-ATP-ase expression and adenylate energy charge in HT-1080 and ZR-75.1 tumour cells also confirmed the altered metabolism. Moreover, the highly limited labelling of citrate by [2-^13^C]-acetate—representing a novel functional test in malignant cells—confirmed the defect of TCA cycle of HT-1080 in contrast to ZR-75.1 cells. Noteworthy, the impaired TCA cycle in HT-1080 cells were associated with high mTORC1 activity, negligible protein level and activity of mTORC2, high expression of interleukin-1β, interleukin-6 and heme oxygenase-1 which may contribute to the compensatory mechanism of TCA deficiency.

**Conclusions:**

The applied methods of energy substrate utilisation and other measurements represent simple assay system using ^13^C-acetate and glucose to recognize dominant bioenergetic pathways in tumour cells. These may offer a possibility to characterise metabolic subtypes of human tumours and provide guidelines to find biomarkers for prediction and development of new metabolism related targets in personalized therapy.

## Background

Following the discovery by Otto Warburg high glucose uptake and lactate production—even in the presence of oxygen—have been documented in nearly all tumours [1, 2]. The glycolytic phenotype was interpreted as a response to damaged cellular respiration to satisfy the ATP demand of the tumours [3, 4]. However, recently numerous studies have showed that ATP production in tumours could be related to both glycolysis and mitochondrion despite of the defected oxidative phosphorylation [4–6]. These intriguing findings received support by demonstrating multiple auxiliary processes for ATP synthesis [6–8].

However, the relative contribution of glycolysis and TCA cycle in ATP production is rather variable in different tumours during progression [4, 5] because the metabolic profile of the tumours depends on both the changes in malignant cells and on the microenvironment [9]. The survival of tumour cells in unfavourable conditions may be attributed to the reprogramming of the metabolism, as a crucial hallmark of the malignant cells [8]. Thus changes of tumour metabolism require individual and regular monitoring. To define the dominant bioenergetic pathway as bioenergetic signature based on the ratio of metabolic markers of glycolysis and mitochondrial respiration has been reported previously [10]. In the present study the bioenergetic signature was estimated by comparing the utilization of glucose and acetate in multiple assays. As acetate is mainly metabolized in TCA cycle contrary to glucose acetate labels only TCA cycle. [11, 12]. Therefore, the comparison of acetate and glucose utilization can be useful to decide the dominant bioenergetic process. Since metabolic reprogramming is directed through regulatory factors it has been suggested that the relevant regulatory mechanisms should also be taken into account [13]. Hence the used complex multiple assay system includes measurements and analysis on substrate utilization and certain related enzymes as the following: (a) measurements on CO_2_ production derived from [1-^14^C]-glucose and [1-^14^C]-acetate; (b) estimation of adenylate energy charge in the presence and absence of glucose and acetate [14]; (c) analyse the expression of metabolic enzymes (G6PDH, GAPDH, β-F1-ATPase); (d) use of stable isotope-mass spectrometric technique to compare glycolysis and TCA cycle activity after labelling tumour cells with [U-^13^C]-glucose or [2-^13^C]-acetate; (e) test the TCA cycle function by measuring the number of ^13^C-carbons in citrate, derived from [2-^13^C]-acetate;.

To gain an insight into the regulation of the aberrant bioenergetic phenotype the expression of mTOR complex 1 and 2 (mammalian target of rapamycin) and of their activity related proteins [15, 16], heme-oxigenase-1 (HO-1) [17] and the proinflammatory interleukins (IL-1β), IL-6, IL-8)) [18, 19] were examined in tumour cell cultures. In contrast to the traditional ^14^C-substrate labelling method, applying the combination of complex assays and measurements help to map the correlations and allow to get more information about the highly different metabolic and regulatory characteristics of cell lines—as it was observed in the two selected cell lines in which the highest difference was detected at their substrate utilization.

## Methods

All materials were purchased from Sigma-Aldrich, except where indicated in the text. HT-1080 (human fibrosarcoma) and ZR-75.1 (human mammary adenocarcinoma) cell lines were selected for detailed characterisation from other human cells (MDA-MB231, BT747—breast and HepG2—hepatocellular carcinoma; Oscort—osteosarcoma, U937—histiocytic lymphoma, isolated fibroblasts) based on their different energy substrate oxidizing capacities.

To obtain subconfluent cultures 5 × 10^5^ HT-1080 and 10^6^ ZR-75.1 cells were seeded in 10 ml RPMI1640 medium with 10 % fetal bovine serum (FBS), 100 IU penicillin and 50 µg/ml streptomycin. In metabolic experiments the medium was replaced by energy substrate-free D5030 medium. All experiments were performed with previously established in vitro cell lines which are not requiring special ethical approvals, The Institutional Tissue- and Cell Culture Laboratory has official permission.

### Measurement of energy-substrate oxidation

Cultures were incubated at 37 °C placed in an air-flow chamber and perfused with CO_2_ free air. Cells were labelled with 0.2 µCi/ml [1-^14^C]-glucose (specific activity 55 mCi/mmol) or [1-^14^C]-acetate (specific activity 57 mCi/mmol (Institute of Isotope-Zrt. Budapest) for 1 h. The CO_2_ released by the cells was trapped on solid alkaline adsorbent, attached to the air-flow chamber and its radioactivity measured with Geiger–Müller counter.

### Expression of mRNA

Total RNAs were isolated with Trizol (Invitrogen). Reverse transcription and real time PCR were performed following the instructions of the manufacturer (M-MLV Reverse Transcriptase kit—Invitrogen; ABI power SYBR^®^ Green PCR Master mix and ABI Prism 7000 Sequence Detection System—Applied Biosystems). PCR primers are summarized in Supplementary Table (ST1). Results were obtained as threshold cycle (CT) values. Expression levels were calculated by using the 2^−ΔCT^ method and β-actin for normalization as reported previously.

### Expression analysis of mTOR complex related proteins, certain metabolic enzymes and inflammatory cytokines at protein level

Proteins extracted from cells were quantitated using Quant-iT protein assay (Invitrogen), separated by SDS-PAGE. Proteins transferred to PVDF membrane were incubated with the following reagents: anti-phospho-mTOR (Ser2448), anti-phospho-S6 (Ser 235/236), anti-Rictor (Cell Signaling Technologies) and anti-β-actin (Sigma-Aldrich), anti-GLUT1 (Abcam), anti-β-F1-ATPase (anti-ATPB Abcam), anti-GAPDH (Serotec), anti-pan-Akt (Cell Signaling Technologies), anti-phospho-(Ser473)-Akt 1 (Abcam) finally with biotinylated secondary antibodies, avidin-HRP complex (Vectastain Elite ABC Kit, Vector) and enhanced chemiluminescence technique (Pierce ECL Western Blotting Substrate).

The concentrations of extra- and intracellular cytokines were determined by cytokine bead assay (CBA immunoassay) were measured by flow cytometry. The assay was carried out using the CBA Human Soluble Protein Master Buffer Kit (BD Biosciences, San Jose, CA, USA; 558264) and CBA Human IL-8 (558277), IL-6 (558276) and IL-1β (558279) Flex Sets (BD Biosciences) according to the manufacturer’s instructions. The samples were acquired on FACSCalibur (BD Biosciences) and analysed by FCAP Array software (BD Biosciences).

### Stable isotope-mass spectroscopic detection of metabolites

Cells incubated in D5030 medium for one hour were labelled with 10 mM [U- ^13^C]-glucose or [2-^13^C]-acetate (Cambridge Isotope Laboratories, Andover, MA, USA). The key metabolites of the glycolytic and TCA cycle pathways were extracted and analysed by applying liquid chromatography–mass spectrometry (LC–MS) based on the protocol described previously [20, 21].

### Determination of adenylate energy charge

To evaluate the distinct contributions of glucose and acetate on the level of adenylate energy charge (AEC) ATP, ADP and AMP were determined in starving cell cultures. To this end the RPMI1640 medium was replaced by the energy substrate deprived medium (D5030) and the cultures were divided into three groups as follows: (a) supplemented with 10 mM glucose, (b) supplemented with 10 mM acetate, (c) kept in starving conditions. Adenylate nucleotides were extracted from the cells with methanol-acetic acid (9:1) were separated by isocratic elution using Aquasil C18 column principally as reported previously [22] and analysed by applying LKB PE NELSON 3000 interface (Perkin-Elmer Corporation).

### Statistical analysis

The experiments were performed in triplicate with 3–5 parallel samples and the data evaluation was performed using Student’s *t* test (two-tailed); *p* < 0.05 was considered statistically significant.

## Results

### Comparing glucose and acetate oxidation

Comparative measurements of released ^14^CO_2_ derived from [1-^14^C]-glucose and [1-^14^C]-acetate showed certain noteworthy differences among the investigated tumour cells. The rate of [1-^14^C]-glucose oxidation was rather similar in the investigated tumour cells, except in HT-1080 cells. On the contrary the release of ^14^CO_2_ from [1-^14^C]-acetate showed great variance especially between the ZR-75.1 and HT-1080 tumour cells (Table 1). The bioenergetic signature calculated from these data indicated that HT-1080,—in contrary to ZR-75.1 cells—preferentially oxidize glucose and have modest capacity to oxidize acetate. Hence this opposite preference for energy substrate has offered promising model to study bioenergetic mechanism in these tumour cells.

**Table 1:** Oxidation of [1-^14^C]-glucose and [1-^14^C]-acetate in human tumour cells measured by the release of ^14^CO_2_

Cell culture	^14^CO_2_ in the cell culture count per 250 s/10^6^ cells		
	[1-^14^C]-glucose (A)	[1-^14^C]-Na-acetate (B)	A/B
HT-1080	11334 ± 2202	377 ± 33	30.1
Fibroblast	3973 ± 684	924 ± 243	4.3
MDA-MB231	3360 ± 584	1344 ± 104	2.5
HepG2	3255 ± 1145	2305 ± 378	1.4
BT747	3081 ± 247	2924 ± 227	1.1
Oscort	5336 ± 171	5580 ± 1240	1.0
ZR-75.1	3450 ± 966	8947 ± 1915	0.4
U937	5948 ± 191	15149 ± 3221	0.4

Tumour cells incubated nutrient-free medium were labelled for 1 h with 0.2 µCi/ml [1-^14^C]-glucose or [1-^14^C]-acetate and the released ^14^CO_2_ measured. Data from three independent measurements and 3 parallel samples

### Expression of enzymes related to bioenergetic pathways

**Fig. 1:**
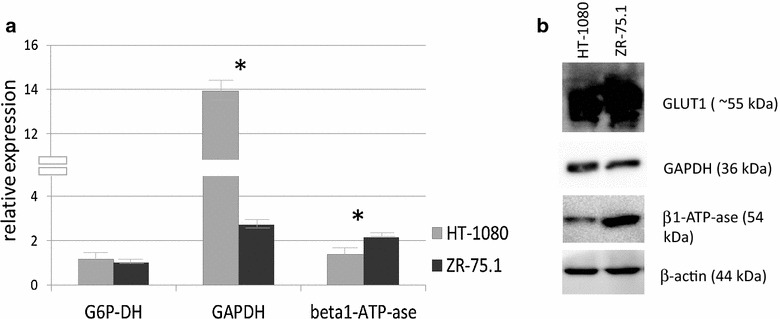
Different expression of certain enzymes related to bioenergetic mechanism at mRNA and protein levels. **a** G6P-DH, GAPDH, β-F1-ATP-ase (beta1-ATP-ase) mRNA expression differences in HT-1080 and ZR-75.1 cells measured by real time PCR (the results as relative expression to β-actin was calculated from threshold cycle values (ΔCT method) of two independent isolation with 3-3 parallels, *- significant). **b** GLUT1, GAPDH, β-F1-ATP-ase (beta1-ATP-ase), β-actin protein expression detected by Western blot in HT-1080 and ZR-75.1 cells

To clarify the background of this some key enzymes related to energy metabolism were studied in HT-1080 and ZR-75.1. The protein expression of GLUT-1 (glucose transporter) showed high level in both cell lines. However, the differences between HT-1080 and ZR-75.1 in glucose/acetate utilization were supported when mRNA expression of enzymes such as glucose-6-phosphate-dehydrogenase (G6PDH), glycolytic glyceraldehyde-3-phosphate dehydrogenase (GAPDH) and mitochondrial β-F1-ATPase were studied. Higher G6PDH (~20 % higher expression) and significantly higher mRNA expression of GAPDH in HT-1080 than ZR-75.1 cells were detected by real time PCR. At the same time mRNA expression of the β-F1 subunit of the ATP-synthase complex was significantly higher in ZR-75.1. The significant mRNA expression differences were confirmed at GAPDH and β-F1-ATPase protein levels by Western blot analysis, as well. In fact this bioenergetic signature, the GAPDH/β-F1-ATPase ratio, is in harmony with substrate oxidation results and confirms the more extensive glycolytic phenotype in the HT-1080 than in the ZR-75.1 cells (Fig. 1).

### ^13^C-glucose labelled glycolytic and TCA cycle metabolites

Applying [U-^13^C]-glucose labelling and LC–MS the highest incorporation of ^13^C atom among the investigated metabolites was measured in lactate both in HT-1080 and in ZR-75.1, noteworthy, that higher level was found in the former cell line. At the same time there was significant difference in the labelling of TCA cycle intermediates between the two cell lines. In HT-1080 cells [U-^13^C]-glucose preferentially donated ^13^C atoms to lactate, G6P and R5P whereas in ZR-75.1 tumour cells malate surpassed the labelling of lactate (Table 2). Thus glucose more extensively fuels TCA cycle in ZR-75.1 than in HT-1080 tumour cells. To study and confirm the importance of the detected distinctions we analysed the metabolic profile of U937 tumour cells, which showed—similarly to ZR-75.1—a relative low glucose oxidizing ability (Table 1). 1-^14^C labelling in glucose/acetate 0.4). The incorporation of ^13^C-carbon from U-^13^C-glucose showed similarity to ZR-75.1 cells. The ratio of labelled metabolites (^13^C-malate/lactate) in U937 was as low as in ZR-75.1 and significantly lower than in HT-1080 (^13^C-malate/lactate ratio- ZR-75.1: 0.87 and U937: 1.17, however, HT-1080: 13.74). Hence the difference in the activity of glycolysis and mitochondrial respiration may be expressed quantitatively by dividing labelled lactate and malate as bioenergetic signature.

**Table 2:** Concentration of glycolytic and TCA cycle metabolites in tumour cells labelled with [U-^13^C]-glucose

Metabolites	ZR-75.1		HT-1080	
	ng/10^6^cells			
	^13^C	^12^C	^13^C	^12^C
G6P/F6P	26.3 ± 2.3	10.1 ± 1.3	72.0 ± 7.1	7.4 ± 1.3
Lactate	39.26 ± 1.6	35.9 ± 6.8	61.2 ± 36.0	70.2 ± 12.6
Citrate	14.9 ± 1.7	33.9 ± 4.8	23.2 ± 4.3	33.9 ± 4.5
Succinate	17.1 ± 7.9	11.8 ± 2.3	ND	ND
Malate	–	28.6 ± 5.9	–	30.2 ± 1.0
	[1-^13^C]:7.9 ± 2.5	–	[1-^13^C]: ND	–
	[2-^13^C]: 22.2 ± 7.0	–	[2-^13^C]: 4.7 ± 0.4	–
	[3-^13^C]: 23.5 ± 7.2	–	[3-^13^C]: 1.9 ± 0.7	–
	[4-^13^C]: 13.1 ± 2.5	–	[4-^13^C]: ND	–

Tumour cell line: ZR-75.1, HT-1080 Culture medium: D5030

Labelling: [U-^13^C]-glucose 10 mM for 1 h. Data from three independent measurements and 3 parallel samples

### Testing the function of TCA cycle

**Fig. 2:**
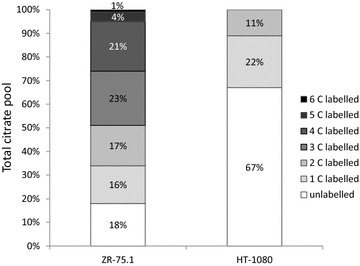
Differences in the number of ^13^C atoms of citrate in ZR-75.1 and HT-1080 tumour cells after ^13^C acetate labelling (% data were calculated from three independent measurements and 3 parallel samples)

As it has been reported that in the mitochondria the second carbon of acetyl-CoA remains in the TCA cycle through several cycling therefore labelling with [2-^13^C]-acetate results increasing number of ^13^C-atoms in citrate. Consequently the enrichment of ^13^C-atoms in citrate represents the intact function of TCA cycle. Applying [2-^13^C]-acetate labelling and analysing the ratio of the unlabelled and labelled, especially the multiple labelled citrate in our samples could indicate the activity of TCA cycle and allow to investigate the cellular TCA capacity deeply and showed the metabolic differences between the two cell lines. In the present study citrates with multiple ^13^C atoms (1–6 number of ^13^C atoms) suggest intact TCA cycle in ZR-75.1, whereas the low number (maximum two) of ^13^C atoms in citrate indicates impaired TCA cycle in HT-1080 tumour cells (Fig. 2). We measured the capacity of TCA cycle in U937 to find correlation in surpassed ^13^C labelling into citrate to ZR-75.1 based on their previously found similarities (Table 1; “^13^C-glucose labelled glycolytic and TCA cycle metabolites” section). The results - the intensively incorporated ^13^C into citrate (1-5 number of ^13^C atoms)—could also confirm the metabolic similarities between ZR-75.1 and U937 (unlabelled citrate 11 %; labelled 1C—19 %, 2C—24 %, 3C—24 %, 4C—19 %, 5C—3 %).

### Effect of glucose and acetate on adenylate energy charge (AEC)

Demonstrating the intact and impaired TCA cycle in ZR-75.1 and HT-1080 tumour cells respectively we focused our interest on the energy level of these cells in the absence and presence of glucose or acetate. The AEC values of HT-1080 and ZR-75.1 cells were rather similar in nutrient rich medium (0.75–0.85) but decreased in the absence of energy substrates (i.e. in D5030 medium), in fact more extensively in HT-1080 cells. However, supplying glucose but not acetate to the starving HT-1080 cells elevation of the AEC value could be achieved. On the contrary in ZR-75.1 cells both acetate and glucose were similarly effective in the restoration of the AEC value. Thus this experimental setting provided further data for more efficient utilization of glucose over acetate by HT-1080 cells (Table 3).

**Table 3:** Elevation of adenylate energy charge (AEC) by glucose and acetate in starving tumour cell cultures

	Adenylate energy charge (AEC*) value	
	ZR-75.1 mammary adenocarcinoma (A)	HT-1080 fibrosarcoma (B)
D5030 medium	0.54 ± 0.15 (100 %)	0.38 ± 0.10 (100 %)
D5030 medium + glucose	0.61 ± 0.15 (113 %)	0.64 ± 0.18 (168 %)*
D5030 medium + acetate	0.69 ± 0.094 (128 %)*	0.46 ± 0.16 (121 %)

$${\text{AEC}} = \frac{{{\text{ATP}} + {1 \mathord{\left/ {\vphantom {1 2}} \right. \kern-0pt} 2}{\text{ADP}}}}{{{\text{ATP}} + {\text{ADP}} + {\text{AMP}}}}$$

Data from three independent measurements and 3 parallel samples

* *p* < 0.05

### Putative modulators of the aberrant bioenergetic pathways

As it has been presumed that reprogramming of bioenergetic mechanisms is controlled by cellular regulation we compared some points of this regulatory network—the expression and/or activity of Akt/mTOR signalling elements, HO-1 and proinflammatory cytokines in HT-1080 and ZR-75.1 tumour cells with the above described different metabolic profiles, TCA cycle activity.

HT-1080 cells showed higher mRNA expression of IL-1β and IL-6 by real time PCR. We could confirm these differences at protein level by cytokine bead assay using flow cytometry. IL-1β and IL-6 proteins were undetectable in ZR-75.1 cells and in their supernatant, as well. According to our PCR results, high intracellular level of IL-1β (159.2 ± 13.2 ng/10^6^ cells) and lower intracellular (81.8 ± 9.5 pg/10^6^ cells) but high extracellular (6.456 ± 0.78 ng/10^6^ cells) level of IL-6 protein were measured in HT-1080. IL-8 —other proinflammatory cytokine—expression was also studied at both mRNA and protein level but such a difference was not detected (IL-8 protein level—7.5 ± 1.3 ng/10^6^ HT-1080 cells; 9.48 ± 1.52 ng/10^6^ ZR-75.1 cells). Moreover, we could detect other interesting differences between these two cells in HO-1 expression level, HT-1080 cells showed higher (more than 15×) mRNA expression than ZR-75.1 cells.

**Fig. 3:**
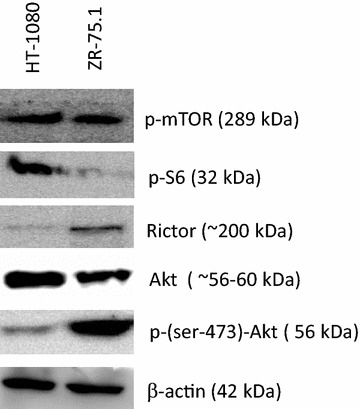
Different expression of mTOR activity related proteins and Akt in HT-1080 and ZR-75.1 cells. phospho-mTOR (p-mTOR—the active mTOR kinase), phospho-S6 (p-S6—mTORC1 activity related phospho-protein), Rictor (mTORC2 specific protein), Akt and anti-phospho-(Ser473)-Akt expression in HT-1080 and ZR-75.1 cells detected by Western blot. The higher amount of p-S6 with low Rictor expression in HT-1080 suggest that mTORC1 activity is high in HT-1080 and lower in ZR-75.1 cells. mTOR activity could be released mainly in mTORC2 complex in ZR-75.1 cells (low level of p-S6 and high level of Rictor and phospho-(Ser473)-Akt)

The further interest focused on mTOR as an important central signalling integrator in regulation of protein synthesis and bioenergetic mechanism. The two mTOR complexes (C1 and C2) represent distinct regulatory signals. In this respect it is noteworthy that different metabolic profiles of the two investigated cells could be associated to distinct expression patterns of mTOR complexes. As Fig. 3 shows HT-1080 cells expressed preferentially mTORC1 activity (represented by high p-mTOR and p-S6—with relative no/low Rictor expression). At the same time in the ZR-75.1 cells showed high p-mTOR with lower p-S6 and higher Rictor level as a sign of potential high mTORC2 expression and activity. Related to mTOR complexes the amount of Akt protein detected by pan–Akt antibody was similar in both cell lines, but higher level of phosphorylated Ser473 form correlates to the higher mTORC2 complex activity and functioning TCA cycle of the ZR-75.1 cells (Fig. 3).

## Discussion

The accumulated evidences on the glycolytic phenotype and repressed mitochondrial function as contributors of tumour progression have initiated attempts to introduce simple but relevant assays to characterise the bioenergetic profile of human tumour samples [23–25]. The majority of studies in this area involves measurements of labelled intermediates in the bioenergetic pathways using radioactive or stabile isotope substrates [21, 25, 26] and different detecting instruments, which represent a great challenge.

Glucose and glutamine are considered the basic and indispensable nutrients subsequently they are the most widely used energetic substrates in metabolic analysis of tumour cells. It has been assumed that using two substrates would provide more information about the relative capacity of glycolysis and TCA cycle than either one alone. As the catabolic product of fatty acid oxidation acetate is channelled toward TCA cycle it appeared to be an ideal complementary energetic substrate beside glucose [27, 28]. Thus acetate can fuel exclusively mitochondrial respiration therefore it is a promising way to detect the bioenergetic mechanism. Some recently published new studies from clinical samples using NMR illustrated that especially acetate could be energy substrate of glioblastomas and brain metastases [29]. In our study we compared acetate and glucose labelling in the substrate oxidation and in the intracellular metabolite concentration analysis by LC–MS. Our results show that using two substrates such as glucose and acetate has more advantage to get detailed information about the relative capacity of glycolysis and TCA cycle. Moreover, our novel evaluation from LC–MS analysis after ^13^C-acetate labelling is capable for highlighting the impairment of TCA cycle in tumour cells. To underline the importance of this metabolic profile analysis we demonstrated some points of the potential regulatory background in the two studied cells, which correlate well to the alterations in bioenergetic phenotype of the cells.

The radioactive technology has still an important role in metabolic investigations including released ^14^CO_2_ measuring, detecting the oxidation of energy substrates offer a simple and cost-effective assay to estimate the dominant bioenergetic mechanism. Applying LC–MS technique with [U-^13^C]-glucose and [2-^13^C]-acetate to investigate tumour specific bioenergetic profile, we demonstrated that the highest level of the incorporated ^13^C atoms derived from [^13^C]-glucose into lactate both in HT-1080 and in ZR-75.1 tumour cells correlates to the other results related to high glucose oxidation and AEC measurement. The LC–MS technique allowed to raise the question whether an identical metabolic phenotype may also be recognized by measuring the unlabelled metabolites which could be interesting in future metabolic analysis of tumour samples. This may have special interest. We found that the unlabelled lactate also reflects to the differences in the glycolytic activity of the two investigated tumours in vitro. However, it should be considered that for example lactate production in tumour cells in vivo is not exclusively derived from actual glycolytic flux (e.g. uptake from microenvironment). The low number of ^13^C atoms in malate and citrate after labelling with [U-^13^C]-glucose or [2-^13^C]-acetate respectively may suggest that TCA cycle is less effective (as we detected in HT-1080 in contrast to ZR-75.1 cells). This provides example for detectable relative dominance of the bioenergetic pathways in tumour cells. The remarkably high unlabelled malate concentration may suggest the function of the anaplerotic route to replenish TCA cycle.

Elevated lactate and reduced concentration of TCA cycle metabolites were described in certain human tumour samples and in patients’ body fluids [30–32] by metabolomics mainly with GC or LC–MS, NMR, but other analytical methods have been developing further. However, there is still no consensual advice in using the analytical techniques to measure metabolic profile of tumour samples (certainly these assays are expensive, time-consuming and need large sample size). In our assays we did not measured the complete metabolite-profile, we only measured and evaluated selected metabolites (G6P, R5P, lactate, citrate, malate, succinate). In that way we could rationalize our sources, we could save time, instrumental capacity and we optimised the sensitivity and apply this methods for smaller sample size. The latter was performed after not only glucose but acetate labelling, as well—the metabolism of malignant cells have not been investigated previously in that way in comparison of acetate and glucose consumption. Upon studying the distribution of ^13^C-carbon, derived from [^13^C-U]-glucose, between glycolytic and TCA cycle intermediates high lactate and low malate labelling indicated glycolytic phenotype and impaired TCA cycle in the HT-1080 tumour cells, but not in the ZR-75.1 cells. Moreover, the highly limited labelling of citrate by [2-^13^C]-acetate—representing a novel functional test—confirmed the above detected defect in TCA cycle of HT-1080 tumour cells. Consequently glucose and acetate differently elevate the reduced level of adenylate energy charge depending on the dominant bioenergetic mechanism.

An other suggestion comparing the expression of glyceraldehyde-3-phosphate dehydrogenase (GAPDH) and β-F1-ATPase an inverse relationship could be concluded between glycolysis and mitochondrial respiration in various human tumour tissues. In fact it was proposed that β-F1-ATPase could be used for monitoring progression of breast cancer [10, 31]. We could confirm this and we can also suggest GAPDH/β-F1-ATPase expression studies in tumour tissue samples by immunohistochemistry based on our Western blot results and its harmony with the metabolic characterisation data.

Evaluating our results from different measurements in HT-1080 and ZR-75.1 cells we could summarise that assays–measuring: (a) ^14^CO_2_ from glucose/^14^CO_2_ from acetate ratio; (b) AEC at the presence of glucose/AEC at the presence of acetate ratio; (c) lactate/malate from ^13^C-glucose labelling; d. the number of incorporated ^13^C into citrate from ^13^C-acetate and e. the GAPDH/β-F1-ATPase expression (summarized in Table 4)—could indicate the metabolic differences at distinct level. From these assays, especially from analysis of the selected metabolites (G6P, R5P, lactate, citrate, malate, succinate) by LC–MS after ^13^C-glucose and acetate labelling we could conclude the condition of mitochondrial function and TCA cycle capacity (impaired TCA or potentially functioning one in HT-1080 or ZR-75.1 respectively) beside the admitted high glycolytic activity.

**Table 4:** Bioenergetic signature based on the utilization of glucose and acetate in various assays

Characteristic data of glycolysis and TCA capacity with different assays	ZR-75.1	HT-1080
^14^CO_2_ from glucose/from acetate	0.4	30.1
AEC at the presence of glucose/at the presence of acetate	0.88	1.39
Labelled^13^C-lactate/^13^C-malate from^13^C-glucose^a^	0.87	13.74
Number of incorporated^13^C atoms into citrate from^13^C-acetate	1–6	1–2
GAPDH/ß-F1-ATPase^b^	1.27	9.81

Summarised data calculated from the results of different assays

^a^The ratio was calculated from molar values

^b^mRNA expression level normalized to β-actin

Our experimental results allowed to find some correlations of certain regulatory factors and the effected bioenergetic pathways or invasive growth in HT-1080 contrary to ZR-75.1 (harbouring less metastatic potential) tumour cells [33]. This expectation was met when high expression of proinflammatory interleukins and HO-1 were detected in the TCA cycle impaired and metastatic HT-1080 cells compared with the non-metastatic ZR-75.1 cells. It was reported that IL-1β and IL-6 stimulate glucose metabolism and promote tumour progression [16]. The high expression of HO-1 in HT-1080 cells deserves special attention as recent studies documented the stimulation of invasive growth and metastatic behaviour associated with high expression of HO-1 in lung cancers (NSCLCs) [17, 34].

At present we may speculate on the possibility that activated rescue mechanism related to HO-1 in TCA cycle impaired HT-1080 tumour cells may enable mitochondrial ATP synthesis through a potential alternative HO-1 dependent way as previously reported in fumarate hydratase 1 deleted kidney cells especially in kidney tumour cells [6].

To study mTOR signalling—which has well documented role in the regulation of metabolic and survival functions—we compared the expression of related phospho-proteins and Rictor in HT-1080 and ZR-75.1 cells [35]. It is well known that malignant transformation is often accompanied by deregulated mTOR activity (mainly higher mTOR activity status) which helps to synthesise and activate the elements/proteins of the cellular apparatus of altered phenotype [36]. The high mTOR activity is providing the enzymatic background for glycolysis, pentose-phosphate pathway and glutaminolysis, moreover the HIF1α production [35, 36]. Present study contributed fascinating data to the distinct function of two mTOR complexes in terms of bioenergetic regulation. The high glycolytic activity with impaired TCA cycle in HT-1080 cells was accompanied by dominant mTORC1 activity, which is in harmony with the reported mTORC1 activity related promotion of glycolysis and the detected GLUT-1 and HIF1α expression. These correlate to the previously described HIF1α expression in HT-1080 and ZR-75.1 at normal condition which could be induced further in hypoxia [37, 38]. On the other hand mTORC2 is rather closely related to oxidative phosphorylation as it is localized in the endoplasmic reticulum sub-compartment which is bound to mitochondria [39, 40]. In this respect it is noteworthy, that expression of mTORC2 complex related Rictor protein was dominant and the related high amount of p-Akt was also detected in ZR-75.1 tumour cells bearing intact TCA cycle. However, the glycolytic capacity and the related GLUT-1 expression, glucose consumption are also not negligible in ZR-75.1 cells.

## Conclusion

In summary, the present study demonstrated the feasibility of novel and cost-effective assays to define the bioenergetic signature based on the comparison of the cellular utilisation of acetate and glucose and to study further specific elements in the regulation of the energetic homeostasis of the cells. The feasibility of this complex energetic profiling assay system (applying minimum two labelling substrates—^13^C glucose and acetate in the measurements of selected metabolites and their ratio and evaluating the number of incorporated ^13^C atoms into certain metabolites—for example into citrate) could define the bioenergetic signature of tumour cells. Moreover, the applied experimental setting provided further evidence for the relationship between the altered phenotype and the impaired/functioning TCA cycle in HT-1080/ZR-75.1 cells. Furthermore the presented complex test system correlated well with the found regulatory differences at protein level—such as mTOR complexes, G6PDH, GAPDH, GLUT-1, β-F1-ATP-ase, HO-1, IL-1ß, IL-6—, and with substrate oxidation/AEC results (Table 4). Based on our observations the presented assays may offer a possibility to characterise metabolic subtypes of human tumours and provide guidelines to find biomarkers for prediction and development of new metabolism related targets in personalized therapy.

## Abbreviations

AEC: adenylate energy charge
CT: threshold cycle
ECL: enhanced chemiluminescence
G6PDH: glucose-6-phosphate dehydrogenase
GAPDH: glyceraldehyde-3-phosphate dehydrogenase
LC–MS: liquid chormatography–mass spectrometry
HO: heme oxygenase
HRP: horseradish peroxydase
mTOR: mammalian target of rapamycin
mTORC: mTOR complex
p-mTOR: phospho-mTOR
pS6: phospho-S6
TCA: trycarboxylic acid
